# Design Considerations for Long Term Non-invasive Brain Computer Interface Training With Tetraplegic CYBATHLON Pilot

**DOI:** 10.3389/fnhum.2021.648275

**Published:** 2021-06-15

**Authors:** Neethu Robinson, Tushar Chouhan, Ernest Mihelj, Paulina Kratka, Frédéric Debraine, Nicole Wenderoth, Cuntai Guan, Rea Lehner

**Affiliations:** ^1^School of Computer Science and Engineering, Nanyang Technological University, Singapore, Singapore; ^2^Future Health Technologies, Singapore-ETH Centre, Singapore, Singapore; ^3^Neural Control of Movement Lab, Department of Health Science and Technology, Swiss Federal Institute of Technology Zurich, Zurich, Switzerland

**Keywords:** tetraplegia, brain computer interface, Cybathlon, electroencephalography, motor assistance

## Abstract

Several studies in the recent past have demonstrated how Brain Computer Interface (BCI) technology can uncover the neural mechanisms underlying various tasks and translate them into control commands. While a multitude of studies have demonstrated the theoretic potential of BCI, a point of concern is that the studies are still confined to lab settings and mostly limited to healthy, able-bodied subjects. The CYBATHLON 2020 BCI race represents an opportunity to further develop BCI design strategies for use in real-time applications with a tetraplegic end user. In this study, as part of the preparation to participate in CYBATHLON 2020 BCI race, we investigate the design aspects of BCI in relation to the choice of its components, in particular, the type of calibration paradigm and its relevance for long-term use. The end goal was to develop a user-friendly and engaging interface suited for long-term use, especially for a spinal-cord injured (SCI) patient. We compared the efficacy of conventional open-loop calibration paradigms with real-time closed-loop paradigms, using pre-trained BCI decoders. Various indicators of performance were analyzed for this study, including the resulting classification performance, game completion time, brain activation maps, and also subjective feedback from the pilot. Our results show that the closed-loop calibration paradigms with real-time feedback is more engaging for the pilot. They also show an indication of achieving better online median classification performance as compared to conventional calibration paradigms (*p* = 0.0008). We also observe that stronger and more localized brain activation patterns are elicited in the closed-loop paradigm in which the experiment interface closely resembled the end application. Thus, based on this longitudinal evaluation of single-subject data, we demonstrate that BCI-based calibration paradigms with active user-engagement, such as with real-time feedback, could help in achieving better user acceptability and performance.

## 1. Introduction

Over the last few decades, several neuroengineering and neuroscience studies have demonstrated how Brain Computer Interface (BCI) technology can uncover the neural mechanisms underlying various tasks and translate them into commands that control an application or device (McFarland and Wolpaw, 2011; Chaudhary et al., 2016; Abiri et al., 2019; He et al., 2020). While many studies have demonstrated the theoretic potential of BCI, especially by deploying novel machine learning methods for detecting distinct task-specific attributes of the brain, a point of concern that remains is that the studies are still confined to lab settings and mostly limited to healthy able-bodied subjects (Lotte et al., 2018). A few case-studies using invasive and non-invasive BCIs have demonstrated the application of BCI as a motor assistive technology for survivors of spinal cord injury (SCI). The reports on BCI applications for tetraplegic patients with SCI include multi-joint robotic arm control using intracortical recordings (Hochberg et al., 2006), cursor control using electrocorticographic activity (Wang et al., 2013), hand orthotic control and wheel chair control in virtual environment using non-invasive electroencephalographic activity (Pfurtscheller et al., 2000; Leeb et al., 2007). The articles (Chaudhary et al., 2016, 2020; McFarland et al., 2020) have comprehensively reviewed the application of BCI as a communication, control, and rehabilitation tool for tetraplegic patients and highlighted that the research in this area is yet to fully evaluate ease-of-use for the end-user and the safe and efficient deployment of BCI in an out-of-the-lab environment.

In recent years, several BCI competitions have been conducted with the goal of providing high-quality brain data to researchers to build effective tools and algorithms that may potentially be deployed in real-world environments (Sajda et al., 2003; Blankertz et al., 2004, 2006; Brunner et al., 2008; Tangermann et al., 2012). The data from these competitions are usually published as open access, allowing researchers to further investigate the brain activity data corresponding to various motor and cognitive tasks. These competitions have served as a means to benchmark the performance of offline BCI and allowed researchers to evaluate and propose novel BCI algorithms by analysing the previously recorded data. Moving forward from offline paradigms, the first Cybathlon was held in 2016 (Novak et al., 2018) included BCI as one of the six race disciplines, introducing an opportunity for benchmarking online BCI systems and to tackle the challenges of a real-world practical assistive application of BCI for people with tetraplegia. The competition succeeded in showcasing BCI to the general public and demonstrating the potential of BCI technology. The second Cybathlon 2020 BCI race (https://cybathlon.ethz.ch/en/event/disciplines/bci) followed a similar format and introduced a multiplayer racing game for the participation of tetraplegic pilots. However, Cybathlon in 2020 took place in front of a virtual audience and the races were recorded asynchronously due to the COVID-19 pandemic. For playing the game, the pilot was required to send correct commands using a BCI within a certain time frame to control the behavior of a virtual avatar. This research article presents a detailed report on the preparation and training of a tetraplegic pilot to participate in the Cybathlon 2020 BCI race. This research investigated the design considerations for a real-time, real-world application of BCI, such as the choice of training paradigms, BCI processing and decoding pipeline, and re-calibration strategies. A longitudinal analysis of the impact of these design aspects on the BCI performance of the pilot is reported in this article. In addition, neurophysiological evidence to supplement the quantitative performance metrics is presented.

A major proportion of BCI literature has focused on improving performance of BCI applications by enhancing the decoding performance of signal processing and machine learning algorithms (He et al., 2020). While this is an important contributing factor, research has also demonstrated that mutual learning of the machine and the user is critical for a successful closed-loop implementation of BCI (Perdikis et al., 2018; Perdikis and Millan, 2020). In any case, the fundamental deciding factor for the efficiency of a BCI system is how well the end-user can generate distinct and consistent brain activity corresponding to each mental task. This in turn results in well-calibrated BCI decoders that can offer better real-time performance. However, the calibration paradigms for data collection, often involve time-consuming, monotonous and non-engaging visual interfaces. Researchers often overlook how well the calibration paradigms elicit the required brain activity in the end-user (Chavarriaga et al., 2017; Roc et al., 2020). The design parameters of the training strategy such as the type of interface used to deliver instructions and feedback and the type of mental task performed by the user are yet to be optimized at a subject level (Roc et al., 2020). Accordingly, one of the primary goals of this research was to investigate the type of calibration and training paradigm that offers better acceptability from the pilot and whether this choice is beneficial for the online BCI performance.

The rules of the game developed for Cybathlon 2020 BCI race stipulated that pilots were required to send three unique control commands to maintain the speed or avoid deceleration of the avatar using their BCI and a fourth control command in which the pilots were required to prevent sending any control signals. This effectively required a BCI that was capable of classifying neural features associated with four mental tasks for which the pilot elicited distinct brain activation patterns. In this research, three motor imagery (MI) tasks were chosen, since they closely associated with the behavior of the avatar, namely, “move left” (left hand MI), “move right” (right hand MI), and “switch headlights on” (both feet MI). More importantly, BCI literature has shown that these MI tasks typically elicit different spatio-spectral patterns of brain activation (McFarland et al., 2000; Neuper et al., 2006; Chaudhary et al., 2016). Thus, an effective decoding algorithm can be used to extract discriminative information from these patterns and use them for classification. In a MI task, the pilot performed a mental rehearsal of the movement without overt motor output. The BCI used in this research was based on electroencephalography (EEG), which non-invasively measures the electrical activity of the brain. The aforementioned power modulations typically occur in the sensorimotor region of brain, termed as event-related desynchronisation/synchronization (ERD/ERS), and characterized by distinct spectral activations time-locked to movement task onset. These features are widely used in EEG-BCI to decode MI (McFarland et al., 2000; Neuper et al., 2006; Pfurtscheller et al., 2006; Nam et al., 2011). Moreover, previous studies in SCI patients have shown that the peak, the area, and the amplitude of the ERD in the mu and beta rhythms were significantly altered compared to healthy participants (Müller-Putz et al., 2014; Foldes et al., 2017). Hence, the typical BCI design required further optimization of the processing pipeline to identify and extract distinct brain activity from the tetraplegic pilot. Cybathlon game rules also required to confirm that the control was achieved solely by using BCI and that ocular or muscular artifacts do not impact performance. Considering the susceptibility of EEG to electronic noise and physiological artifacts arising from sources other than the brain, such as eye movements (He et al., 2020), the BCI design also consisted of pre-processing, including a real-time artifact detection and correction.

The rest of the paper is structured as follows. In section 2, the details of study are presented including the training strategies, components of BCI, and implementation of closed-loop BCI for Cybathlon preparation, training, and final event. The research outcomes of this study, including quantitative and qualitative evaluation of BCI performance and analysis of the efficacy of different calibration paradigms are presented in section 3. In section 4, the discussion of the results are presented focusing on the challenges and research avenues for future research that may enhance the feasibility of a real-time real world application of BCI for a tetraplegic patient.

## 2. Materials and Methods

### 2.1. Ethics Statement and Recruitment of the Pilot

All the protocols and procedures in this study have been approved by the ETH Zurich Ethics Committee (EK 2019-N-01). The inclusion criteria were aligned with those of the Cybathlon 2020 BCI race. The latter stipulated that the result of the formal neurological examination using the American Spinal Injury Association (ASIA) International Standard for Neurological Classification of Spinal Cord Injury (ISNCSCI) must correspond to a neurological level of injury of C5 or above (i.e., a spinal cord injury with impairment at and below the neck) as well as an ASIA Impairment Scale (AIS) of A, B, or C. At least three out of five key muscles in each extremity must have a muscle function grading below 3 (i.e., no antigravity muscle strength). The exclusion criteria consisted of cyber-sickness, epilepsy or similar problems. Under these criteria, the pilot recruited for this study is a male left-handed young adult who suffered a spinal cord injury 6 years prior to the experiment at the neurological level of C4. The pilot scores B on the ASIA impairment scale meaning there is some sensory, but no motor function preserved below the neurological level. He did not have any previous experience in BCI experiments prior to this research. All the data reported in this study were recorded either at the pilot's home or at Neural Control of Movement Lab at ETH Zurich.

### 2.2. Cybathlon 2020 BCI Race

A multiplayer computer racing game, named BrainDriver was developed for the BCI Race in Cybathlon 2020. Seven tetraplegic pilots competed asynchronously against each other and used a BCI to control the behavior of a virtual avatar moving along a virtual track. During the race, the pilots sat in front of their respective screens and observed their avatar on the track. The virtual race track was divided into dedicated zones (tasks), indicated by road signs or lines on the ground and the pilots were required to send appropriate commands using their BCI within the correct time frame. Once the race started, each avatar moved forward by itself toward the finish line of the race, by default.

Pilots were allowed to send three different commands to control their avatar. Sending the appropriate command at the right time was required to maintain the avatar's speed, while wrong input or no input (if input is required) slowed down the avatar. Pilots could trigger their avatar to turn left (LEFT) or right (RIGHT). In case of sudden changes in the environment, i.e., streetlights turning off, the pilots were required to react with the appropriate BCI input (HEADLIGHT). In certain parts of the game, no signals were to be sent (NOINPUT) and avatars decelerated if they received any command by accident. LEFT, RIGHT, and NOINPUT commands could be anticipated by the pilot, whereas the HEADLIGHT signal had to be generated in response to a changing environment. The game covered a virtual distance of 500 m and each track condition appeared four times in total. The pilot who crossed the finish line first, or the pilot who covered the longest distance within the race time limit of 4 min, was declared as the winner. In compliance with COVID-19 pandemic restrictions, the format of Cybathlon 2020 BCI was modified such that each pilot/team competed individually from different locations. The pilot played three runs of the game during a 3 h time window. The best game finish time out of the three was counted toward the final ranking. All three games were recorded and refereed by the Cybathlon organizing committee.

### 2.3. Experiment Set-Up and Training

**Figure 1:**
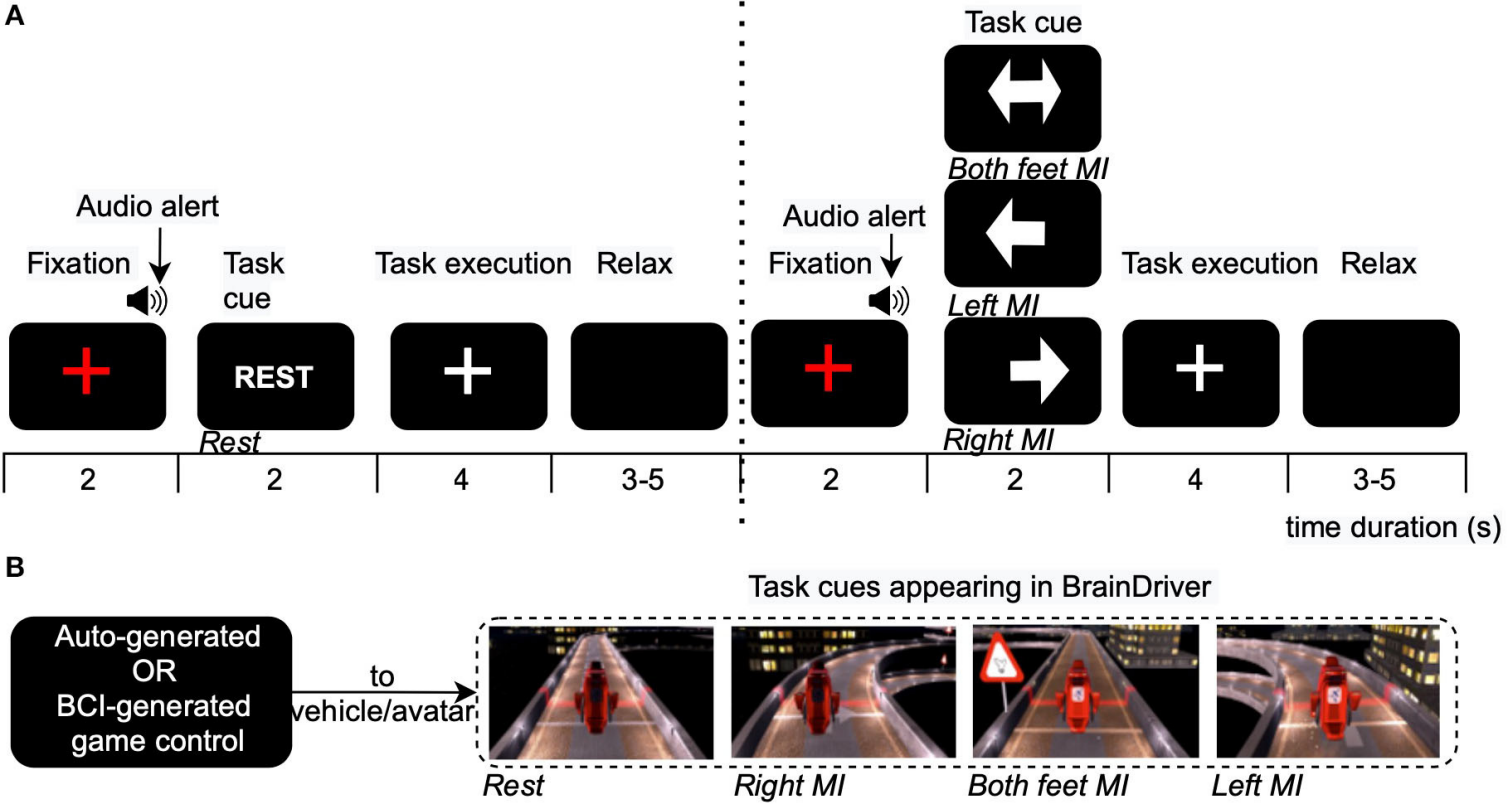
Timeline and experiment interface under different calibration paradigms. **(A)** Arrow-based calibration paradigm (*aC*): The conventional MI-BCI interface is modified to include a “REST” trial prior to every motor task. The visual cues presented to the pilot and the associated instruction is illustrated. **(B)** Game-based calibration paradigm (*gC*) and game-based evaluation paradigm (*gE*): The BrainDriver interface is used to deliver visual cues. In *gC*, the game control is automatically generated by a program which replicates the expected input to control the game accurately. In *gE*, the BCI controls the game in real-time. In both these paradigms, the pilot is instructed to generate relevant brain activity to play the game. The white horizontal line across the track, in the snapshots, depicts the beginning of the next MI zone.

**Figure 2:**
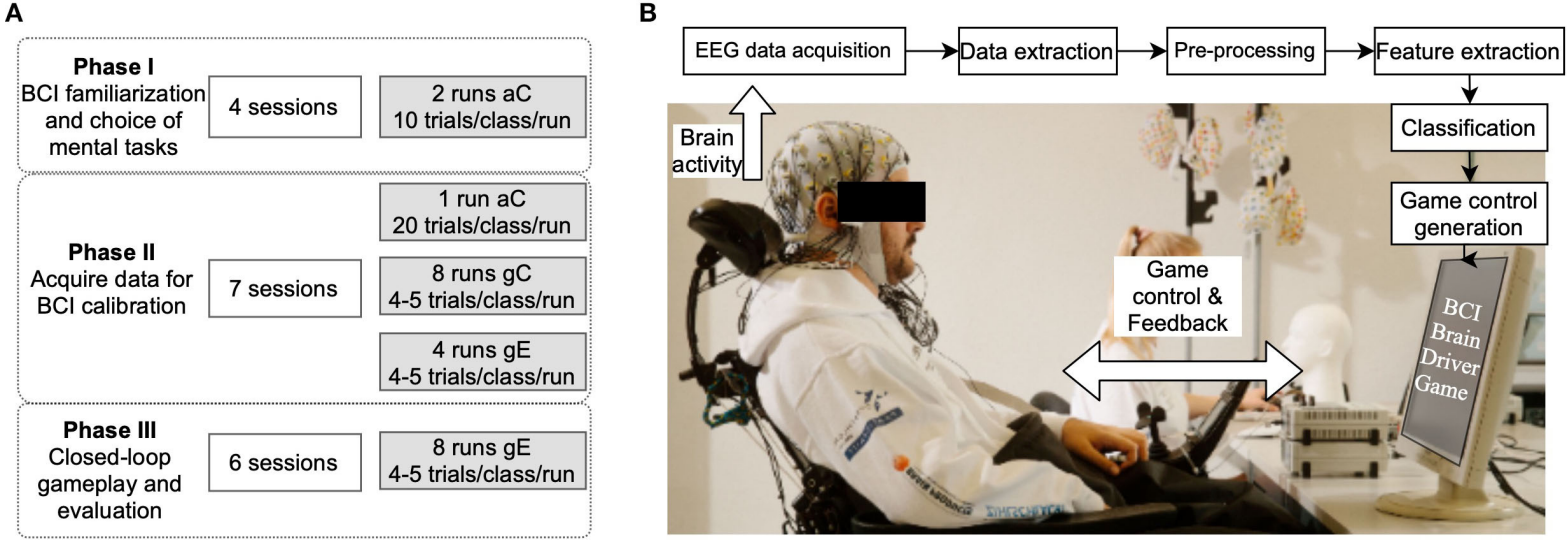
Overview of experiment. **(A)** Strategy: summary of the training sessions that were analyzed in preparation to participate in Cybathlon. The objective of each experiment phase is indicated along with the number of experiment runs across the training sessions. **(B)** Setup: BCI facilitates a closed-loop interaction between the pilot and the game interface. The brain activity generated by the pilot as he performs mental tasks are translated to commands to control the movement of a virtual avatar in the game.

The key elements considered in the design of a BCI experiment and its implementation as a closed loop application were the BCI processing pipeline, calibration paradigms, and data acquisition. The recorded EEG data were then used for calibrating the BCI and optimizing the training strategy for the pilot to control the BCI. The calibration paradigms investigated in this research consisted of both open-loop and closed-loop implementations of BCI. The paradigms were evaluated for the efficacy in eliciting distinct task-specific brain activity as well as for their engagement of the pilot. The processing pipeline implemented in this study was a conventional multi-class BCI decoder in which each component was fine-tuned to optimize the decoding performance. The duration of entire study was 1 year and 10 months, and the pilot was trained from May 2019 to March 2020 and July 2020 to Oct 2020. The interruption in training period was due to the restrictions imposed by COVID-19 pandemic protocols. An overview of each of these elements are illustrated in Figures 1, 2 and their details are discussed further in the following sub-sections.

#### 2.3.1. Calibration Paradigms

The traditional calibration paradigms used in motor BCI systems involves an open-loop BCI design with a graphical user interface (GUI) that presents instructions as audio-visual cues (Pfurtscheller and Neuper, 2001). Here, the BCI performance entirely relies on the subject's ability to generate neural commands by voluntarily initiating and consistently carrying out the instructed motor tasks. There is still a lack of understanding of how the factors such as presentation, duration, presence of feedback etc., impact the pilot's learning and control of BCI (Roc et al., 2020). However, a general consensus is that to ensure user participation, the interface needs to be engaging as well as resemble the end-application. This has also been also suggested by recent works (Škola et al., 2019; Roc et al., 2020). Keeping this in mind, the research presented in this paper investigated three different modes of calibration paradigms and evaluated the pilot's BCI decoding performance, subjective feedback, and brain activation patterns in each case. As mentioned earlier, the tasks used to generate control commands were relaxation, MI of left hand and right hand for three of the four control commands. These MI tasks were chosen since they closely relate to the behavior of the virtual avatar in the game and have been seen to elicit different brain activation patterns underpinning the corresponding neurophysiological mechanisms (McFarland et al., 2000; Neuper et al., 2006). For the fourth control command, we investigated two options: both feet and both hands, to identify the MI class that would result in better overall classification performance. The pilot was instructed to imagine self-paced clenching of either hand and pedaling movement of feet for the respective motor tasks.

The most commonly used BCI calibration paradigm is the synchronous, open loop BCI that presents text-based or image-based cues to indicate the expected motor tasks. Following this, an experiment interface termed as arrow-based calibration, (*aC*) was used in this research. The display screen associated with each instruction across the timeline for *aC* is presented in Figure 1A. The display on the left and right side of the dotted line indicated a “REST” and motor task trial, respectively. The motor task could be one of the three MI tasks used in this study, and as indicated, a “REST” trial preceded each MI trial. The start of each task trial was indicated by a red cross, and an audio alert was played before every task cue. The task duration was 4 s, and the pilot was instructed to fixate his eyes on the white cross at the center of the screen while performing the task. At the end of the task, the cross disappeared and the pilot was allowed to relax until the screen remained blank.

The research also investigated the use of BrainDriver game interface for pilot training and acquiring calibration data. Figure 1B indicates the display of the game and how each task was associated with the game environment. The game may be interpreted as a randomized and repeating combination of four types of MI zones described in section 2.2. As can be seen in Figure 1B, a white horizontal line across the track depicted the beginning of an MI zone. Two types of MI zones corresponding to the right and left turns of the virtual track were controlled by the pilot by performing right and left hand MI, respectively. The caution sign and turning off of the headlight served as the cue for both hand or both feet MI (third type of MI zone). The no input sign on the track cued relaxation (fourth type of MI zone). To enable continuous generation of correct control commands, the pilot was asked to start performing the task once the virtual avatar/vehicle crossed the white line on the track and keep performing MI for the entire duration of zone. Thus, a continuous stream of control commands were provided to the game throughout the entire duration of the race. To avoid any learning effect on the pilot, the race track for each game was randomly generated prior to each recording. Thus, the pilot remained unaware of the order in which tasks needed to be performed to control the game. The research first examined an open-loop implementation of this interface, in which the control of the virtual avatar was generated by a computer program, which always accurately generated the expected control. This was termed as game-based calibration (*gC*). The interface in *gC* was visually more engaging than *aC* and allowed the pilot to familiarize with the end application.

The drawback in both approaches explained above was the lack of presentation of feedback in the interface. A feedback can represent the BCI detection accuracy of the brain activity generated by the pilot and can act as an incentive for the pilot for better engagement, regulation and control. Thus, the research also investigated a closed-loop implementation of BCI-BrainDriver game in which the behavior of the avatar was controlled by the pilot using the BCI. This interface was used in the final evaluation as well, and hence, was termed as game-based evaluation (*gE*). Compared to open-loop designs, *gE* ensured the involvement of the pilot, and allowed him to self-regulate his brain activity to improve performance. However, this approach required a pre-calibrated BCI decoder whose performance may be a bottleneck in collecting high quality brain data.

#### 2.3.2. Training

This paper features a longitudinal evaluation of the BCI training of a single tetraplegic participant. The impact of each calibration paradigm and training strategy on BCI performance, and how they vary across different experiment sessions were further investigated. The entire training period was divided into three phases as indicated in Figure 2A. In phase I, the conventional *aC* paradigm was used to record data in which the pilot executed different MI tasks. The data collected was then analyzed offline to select the motor tasks that were used for the rest of the study. In phase II, the protocol consisted of calibration (*aC, gC*) and evaluation (*gE*) runs. The data from phase II was analyzed retrospectively to determine the training strategy for phase III. The data from phase II was also used to train a BCI classifier model which was used in phase III. In phase III, the protocol consisted of only *gE* runs. The objectives of each training phase and the experiment details are explained in the following sub-sections. The training period ended 2 weeks prior to Cybathlon, after which the pilot participated in a pre-finals practice race and the Cybathlon finals. The performance of the subject across the entire training period and Cybathlon was tracked and reported in section 3.1. The data collected was used for further analyses as explained in section 2.3.3.4.

##### 2.3.2.1. Phase I: BCI Familiarization and Selection of Tasks

The primary objective of this phase was to introduce the BCI system to the pilot, familiarize him with both offline and online BCI setups as well as the BrainDriver game and the execution of MI tasks. The second objective was to identify the motor tasks that help to attain better BCI performance for the pilot. The three tasks namely right and left hand MI and rest were fixed throughout the experiment, however, the fourth task was chosen based on pilot's feedback on the ease-of-execution, the offline evaluation of brain activation patterns and decoding performance. This phase lasted from May 2019 to January 2020, and the pilot participated in 10 experiment sessions during this time.

As indicated in Figure 2A, only four out of the ten recorded sessions were used in the offline evaluation and further analysis. Of the remaining sessions, five had to be discarded from the final analysis due to technical issues (e.g., malfunctioning of triggering device) and one session was discarded due to too much distraction in the recording environment. The standard calibration interface, *aC*, was employed in the four sessions chosen for further analysis. Out of these four sessions, the pilot performed both hand MI for the first two and both feet MI for the next two sessions, during the third motor task trials. As indicated in the figure, the pilot participated in two runs of experiment in every session. In each run, 10 trials of each of the MI tasks and 30 trials of rest were recorded. During this phase, a few practice sessions were also conducted to allow the pilot to familiarize himself with the BrainDriver game. In these sessions, game control was delivered partially by a BCI and partially by a computer programme.

##### 2.3.2.2. Phase II: BCI Calibration and Pilot Training

The objective of this phase was to investigate and identify the calibration approach that offered the best user engagement and helped to acquire data with the best decoding performance. This phase spanned from February to March 2020 and July to September 2020 and consisted of seven experiment sessions that followed a standard paradigm as follows. In every session, the pilot participated in one run of *aC*, followed by eight runs of *gC*. The recording lasted for about 40 min per session. The *aC* yielded 20 trials of data belonging to each MI task and 60 trials of data belonging to rest task. Each run in *gC* yielded four trials of data in each class. Following the calibration, the pilot participated in four runs of online game play, *gE*. A BCI model was calibrated using the data collected during *gC* and was used in these four game runs. The game finish time (*τ*_*finish*_) for each *gE* run was also recorded.

##### 2.3.2.3. Phase III: Evaluation of BCI

The final phase of training focused on allowing the pilot to learn to control the BCI and hence only the closed-loop game interface *gE* was used. This phase lasted for 2 months (October to November 2020) and included six sessions. A BCI classifier model was pre-calibrated using the entire data collected, i.e., data from *aC, gC*, and *gE* from all sessions, during the previous phase. In each session, the pilot performed eight runs of *gE*. The game control in the first four runs was generated by the pilot using this pre-calibrated BCI. A new BCI model was then calibrated using the data from the first four runs of *gE*, which yielded 16 trials per task. This model was then used to generate game control in the last four runs of the session. This training strategy was followed for all six sessions in phase III.

The game performance and the offline analysis of data from phase II and phase III indicated that a BCI calibrated based on multiple-sessions of *gE* offered the best performance for the pilot; and the performance improved further when the BCI is calibrated using data from *gE* recorded immediately prior to the evaluation game. Hence, a BCI model was calibrated using all the recorded data from phase III and was used in both the pre-finals practice session and the final session. The practice session was conducted on the day prior to Cybathlon finals. For these two sessions, similar to phase III, the pilot participated in four runs of *gE*, that used pre-calibrated BCI model from phase III for game control. A new BCI model was then calibrated using these four runs. Subsequently, the pilot engaged in three game runs using this new BCI model.

#### 2.3.3. BCI Processing Pipeline

In this study, EEG was recorded using a BrainVision 64-channel actiChamp amplifier with actiCap active electrodes. The EEG was recorded at a sampling rate of 500 Hz. From phase II onwards, four electrodes from the actiCap were used to record vertical and horizontal electrooculographic (EOG) activity. One pair of electrodes was placed below and above the right eye (vEOG) and the second pair (hEOG) was placed at the outer canthi of the eyes using adhesive tapes. For the analysis and classification, a 4 s data segment was extracted from the acquired multi-channel data. In the offline mode, 4 s segments synchronized to the task onset were extracted. In the online mode, at regular intervals of 1 s, a segment containing the last 4 s of data was extracted from the EEG datastream. The recorded data belonged to one of the four distinct tasks and the data segments were labeled accordingly as ω∈ {Left MI, Right MI, Feet MI, Rest}. Since the pilot was asked to keep performing the corresponding MI continuously within an MI zone, the classified labels from the extracted time windows, described above, were then compared with the correct labels that were required by the game to measure classification performance. This section details the signal processing and machine learning components of BCI that were employed in the translation of the acquired and extracted brain activity to game control commands.

##### 2.3.3.1. Artifact Removal

The Cybathlon BCI race regulations required a mandatory artifact rejection or correction approach to ensure that the game control was not influenced by ocular activity. To incorporate this, the proposed EEG signal processing pipeline started with an artifact removal module, specifically designed to detect and correct eye movement artifacts in real-time. The first step in this approach was to detect whether an EEG channel was corrupted by vertical or horizontal movement of the eyes. For every 4 s segment of EEG, in both offline and online modes, the correlation of EEG channel data with the four EOG channels in terms of Pearson's correlation coefficient was computed. The EEG channels were then sorted according to the maximum average absolute correlation with the vEOG and hEOG channels. Two EEG channels with the highest correlation were then marked for artifact correction.

**Figure 3:**
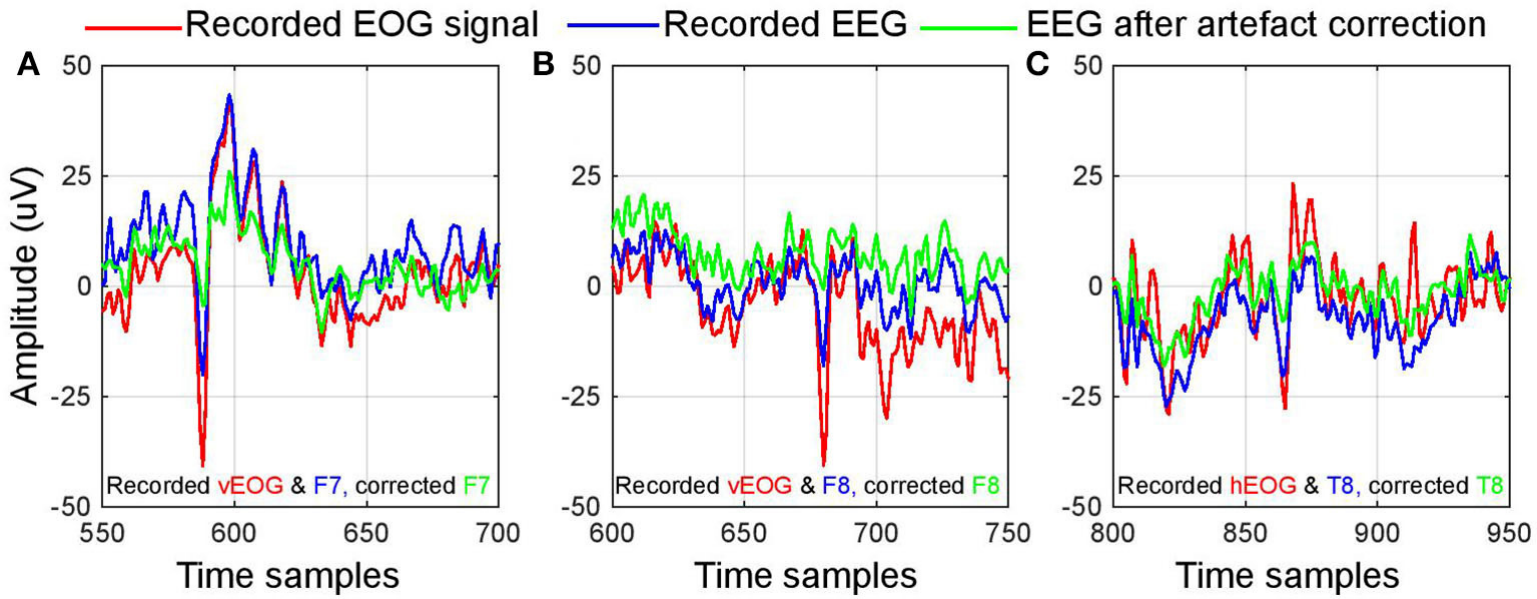
Demonstration of artifact removal. **(A–C)** Illustrates three different instances of artifact correction employed in the BCI used in this research. The blue line indicates a segment of data that the algorithm identified as corrupted by ocular artifact and the red line indicates the simultaneously recorded electrooculugram (EOG) signals. The green line displays the same segment after correction, with visibly reduced EOG peaks.

The data from the corrupted channels were then removed and interpolated using the remaining EEG channels. The interpolation was carried out using spherical splines, which is a typical artifactual signal replacement tool in EEG analysis (Perrin et al., 1989). This approach was defined to suit both offline and online BCI designs, and offered fast and efficient execution without the need for pre-trained prior-data dependent model. Figure 3 illustrates three data segments of EEG (blue plot) that the algorithm automatically detected as corrupted by EOG (shown in red). The data that was interpolated from the remaining channels is indicated by the green plot. It can be observed that, after artifact correction, the amplitude of artifact peak in EEG channel is considerably reduced, whereas the rest of the segment remains minimally affected.

The artifact removal was followed by low pass filtering of EEG at 40 Hz using a Chebyshev Type II filter. This step removed high frequency noises and powerline interference at 50 Hz. The preprocessed data was then passed on to the next step for feature extraction.

##### 2.3.3.2. Feature Extraction and Classification

The BCI used in this study employed the classical feature extraction algorithm Filter Bank Common Spatial Pattern (FBCSP) (Ang et al., 2008, 2012) which is a widely used algorithm for benchmarking EEG datasets. FBCSP was originally reported for the classification of 4-class MI (left hand, right hand, feet, and tongue) data and was the winner of BCI Competition IV Dataset 2a. The algorithm comprised multi-band spectral filtering of EEG data, followed by spatial filtering by Common Spatial Pattern (CSP) algorithm in each frequency band. The features were then derived as log-variance of spatially filtered signal. A subset of features were selected based on mutual information, which were then sent for classification. In this study, the parameters of FBCSP (such as number of CSP filters) were fine-tuned and modified for a multi-class implementation using data from phase I. A one-vs.-one approach was employed in which the FBCSP filters were computed for *k* = 6 binary combinations of four classes of data. The features were then accumulated and fed to a Support Vector Machine (SVM) classifier with a Gaussian kernel. The procedure for calibration is summarized in column 1 of Algorithm 1.

The first step in FBCSP algorithm was multi-band (*f* = 9) filtering of preprocessed EEG using Chebyshev Type II band-pass filters. The frequency ranges were 4–8, 8–12,., 36–40 Hz. The next step computed a CSP projection matrix, *W*_*k*_ that transformed the EEG data in each band so as to increase the discrimination in terms of the signal variance between two classes. The feature vector, *F*_*k*_ was derived as the normalized log-variance of the CSP filtered data from the first and last *m* = 2 columns in each band. For the classification of features a SVM classifier was employed, using the LIBSVM toolbox (Chang and Lin, 2011). For each *k*, a classifier was trained as *C*_*k*_.

##### 2.3.3.3. Online Engine and Feedback

A closed-loop design of the BCI was implemented to deliver real-time feedback to the user by translating the EEG activity to game control commands. From the multi-channel EEG datastream, every 1 s, a 4 s segment of data was extracted, *X*_*t*_. This data segment was passed through the data processing steps mentioned in the above sub-sections and converted to a feature vector, *F*_*t*_*k*__. A set of classifier outputs were determined by each of the *k* = 6 binary classifier as ${\hat{y_{t}}}_{k}$. The final output was determined by a simple voting of class labels as $\hat{y_{t}}$ (Chang and Lin, 2011). The steps are summarized in column 2 of Algorithm 1. The BCI output was mapped to one of the four game controls and was sent to the BrainDriver game.

##### 2.3.3.4. Offline Analysis

In order to evaluate the various design parameters used in the final BCI, a series of offline analyses were performed on the recorded data. The objectives and steps involved in these analysis are listed below.

*Cross-validation analysis*: This study investigated the type of calibration interface which efficiently engaged the user to elicit more discriminative, task-specific brain activation patterns. To evaluate this, the classification performance of data collected under the three different experimental interfaces (*aC*, *gC*, and *gE*) was determined by a 10-fold cross validation. Before the analysis, the dataset was balanced to ensure that equal number of trials were taken from each class ω. This was achieved by randomly selecting the same number of trials for each class as the class with the lowest number of trials. Typically, the “REST” class contained more trials than the other classes. Therefore, a subset of trials from this class were randomly picked to match the sizes of the other classes. The average kappa value across 10-folds, *κ*_*cv*_ was used as the performance metric to quantitatively assess the efficacy of the calibration paradigms.

**Algorithm 1:** Calibration and evaluation.

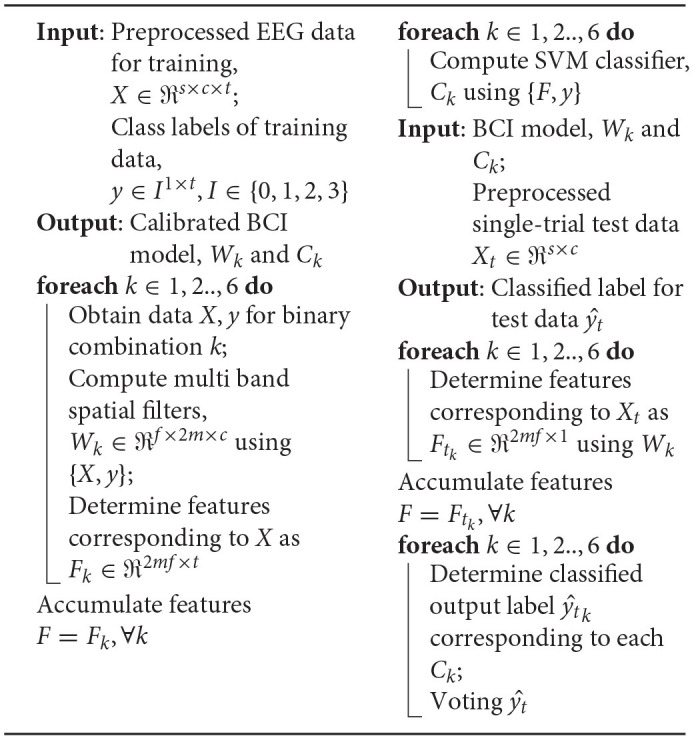

*Pseudo-online analysis*: In this analysis, the closed-loop BCI implementation was replicated using the data recorded during closed-loop testing. The objective was to compare the online performance of different BCI calibrated models on the same data stream. The kappa value *κ* was used as the quantitative metric of the pseudo-online BCI performance.

A quantitative assessment of the efficacy of different calibration paradigms was carried out using *κ*_*cv*_ and *κ* and were then subjected to the Wilcoxon Rank Sum test. These metrics indicate how well the calibration interface elicits distinct task-specific brain activity from the pilot, in terms of the detection of tasks within the same run (*κ*_*cv*_) and the online BCI performance on a separate game run (*κ*). The interface with higher performance might be more suitable for a self-motivated calibration in a practical application.

##### 2.3.3.5. EEG Data Analysis

*Analysis of Both Hands vs. Both Feet MI*: Phase I of our training shared three MI classes (Left hand, Right hand, and “Rest” class) for all sessions, while they differed in the fourth class (Both hands vs. Feet class). Thus, we sought to investigate which choice of the fourth class was more dissociable from the remaining three classes, so we could make an informed decision on which class to continue the training process with. Although the overall classification performance, as measured by *κ*, was the key performance metric for this comparison, we also sought to further assess the discriminative ability of the underlying neural features associated with these classes. To this end, we employed representational similarity analysis (RSA, Nikolaus Kriegeskorte, 2008) assessing the pairwise distances that Both hands class and Feet class exhibited when compared to other three classes. For this analysis, we used the bandpowers in the mu and beta bands to compute the distances associated with the mutivariate patterns of each class. The following steps were undertaken. The preprocessed data were band pass filtered within the known sensorimotor frequency range (8–30 Hz: mu and beta bands). The band powers from the preprocessed and filtered data (between 8 and 30 Hz) were averaged across all trials within a particular class. Standardized Euclidean distances were then calculated for each of the four sessions of phase I in the following way. We started by comparing the pairwise distances between the Both Hands class, and the remaining three classes (i.e., Both hands vs. Left, Right and Rest), with the pairwise distances between Both Feet class, and the remaining three classes (Both Feet vs. Left, Right and Rest). This resulted in four representational dissimilarity matrices, each representing all the pairwise distances between the classes. Finally, distances relating the Both Hands and the Both Feet classes vs. the remaining three classes were extracted and statistically compared. Larger distances indicated greater separability of that class from the remaining three classes.

*Comparison of Calibration Paradigms*: In phase II, we first endeavored to find neurophysiological evidence of more discriminative information elicited as response to the proposed game-based calibration paradigm *gC* and the online games with real feedback as compared to *aC*. This would then provide us with motivation to select a more effective paradigm for phase III. For this purpose, we compare the efficacy of *aC* and *gC* in producing discriminative brain activation patterns which are necessary to effectively differentiate between different MI classes. For *aC*, this corresponds to the task execution period, as shown in Figure 1. For *gC* and the online games, a 4s time window immediately following the onset of an MI zone was extracted. As can be seen in Figure 1, the onset of a new MI zone was marked by a white horizontal line across the virtual track. Therefore, the EEG data corresponding to the time instant when the avatar crossed this line was used to mark the beginning of the 4 s time window. We also compare the brain activation patterns of the online games and compare them to those elicited by *aC* and *gC*, to demonstrate the motivation behind using the games with real-time feedback as our calibration paradigm in phase III (*gE*). This is done by comparing the relative power of EEG data in the mu EEG frequency band (8–12 Hz), which is computed as the absolute power of the mu band normalized by the wideband power in the preprocessed EEG data low pass filtered at 40 Hz (Neuper et al., 2006), across the three paradigms in phase II and visualized as a head topoplot. For the online games, the time window used for this analysis was extracted similar to that of *gC*. Since we use a FBCSP-based model, we expect that a certain paradigm eliciting greater discriminative brain activations would show more localized information around the typical sensorimotor regions of the aforementioned relative power patterns obtained from EEG (Neuper et al., 2006). Diffused patterns, on the other hand, may result in inferior classification performance.

Table 1 presents a summary of the usage of data collected during phase I, II, and III under multiple runs of *aC*, *gC*, and *gE* paradigms for the aforementioned analyses. The results of these analyses are reported in the next section.

**Table 1:** Summary of the usage of data for classification and data analysis.

		**Phase I**	**Phase II**			**Phase III**	
**Analysis**		**aC**	**aC**	**gC**	**gE**	**gE**	**gE**
	Cross-validation analysis						
	Calibration						
Performance of calibration paradigms	Online evaluation						
	Calibration						
Impact of recalibration	Online evaluation						
Analysis of brain activation patterns							

## 3. Results

The main aim of this work is to investigate the effectiveness of using a closed-loop calibration protocol in an online BCI system over a conventional, open-loop calibration protocol. However, before proceeding to the results, we first present some preliminary analyses to identify the most suitable metrics to evaluate the performance of different calibration protocols. Thus, this section presents the BCI performance of the pilot across multiple sessions in the training period and the results of the offline analysis. The study is designed toward preparing the pilot to participate in Cybathlon 2020 BCI race. Therefore, the primary outcome of the study is performance in terms of game finish time (*τ*_*finish*_), which is inversely correlated with classification performance of the BCI, as expected. The pilot participated in several online game sessions across the training period and the variations in *τ*_*finish*_ are presented in this section. This research investigated the design considerations for a practical BCI system and conducted a longitudinal evaluation of BCI performance under different calibration paradigms. The results of the quantitative assessment of the various elements of calibration paradigms such as the calibration interface and the frequency of calibration are reported in this section.

### 3.1. Game Finish Time and Cybathlon Performance

**Figure 4:**
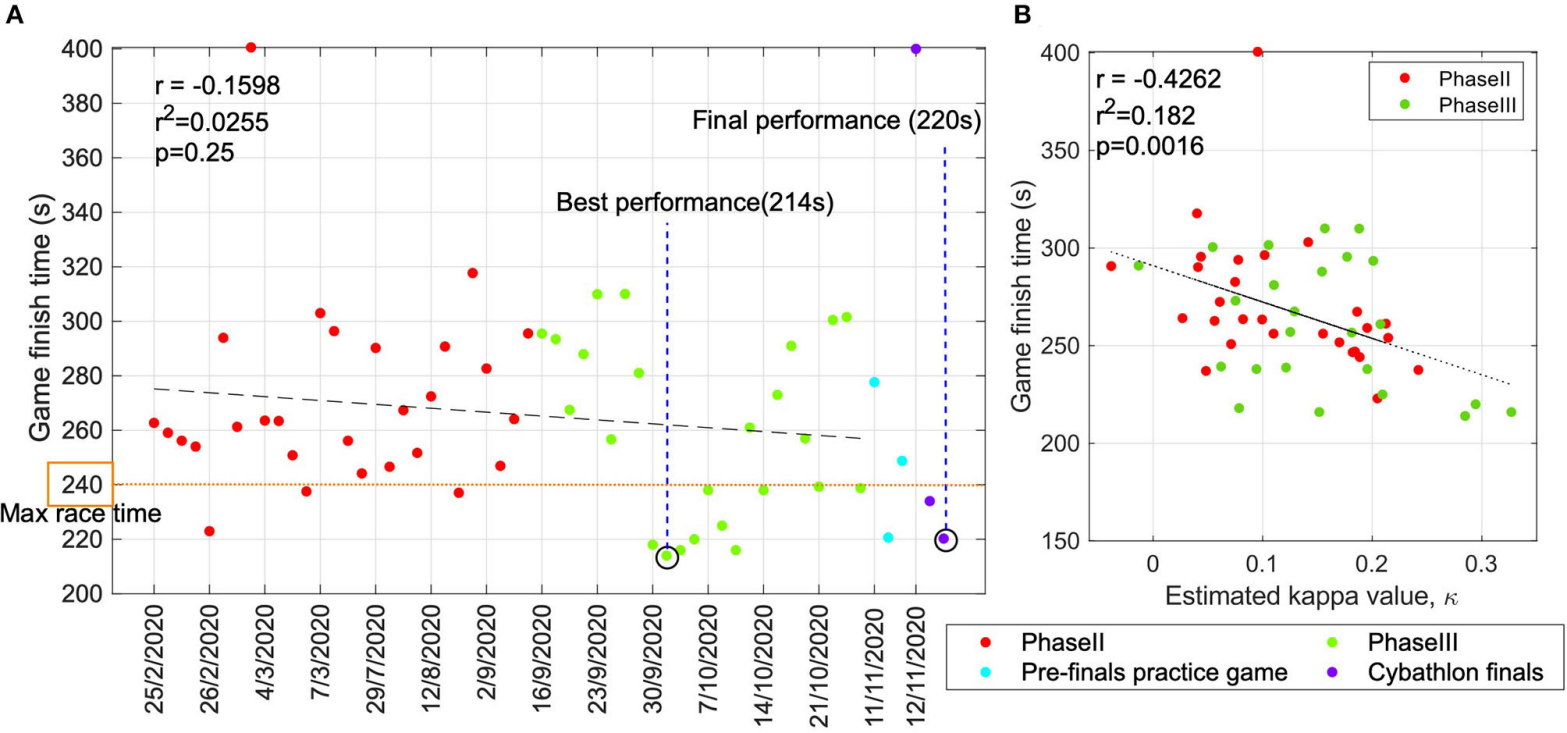
Performance of the pilot across the training period and Cybathlon. **(A)** Cybathlon BCI race required the pilot to navigate a virtual distance of 500 m in under 240 s. The game finish time, *τ*_*finish*_ achieved by the pilot throughout the training is shown in this figure. The black dashed line indicates the linear fit and the qualifying time is indicated by the horizontal orange dashed line. The performances in phase II, phase III, pre-finals, and final Cybathlon race are indicated by red, green, blue, and purple markers, respectively. **(B)** The relationship between performance metrics *κ* and *τ*_*finish*_ across the training phases II and III. Sessions with higher decoding performance, *κ* record shorter race finish time, *τ*_*finish*_. The black dashed line shows the linear fit and the significant *p* = 0.0016 inverse relation between the two metrics.

In this section, we present the results of training, in terms of the game finish time, across the entire training period. In Figure 4A, the red and green markers indicate the performance in *gE* runs from phase II and phase III of training, respectively marked chronologically on the *x*-axis. The blue markers indicate the performance in three runs during a pre-finals practice game and the purple markers indicate the three runs during the Cybathlon finals. The qualifying time of 240 s, as per the rules of Cybathlon BCI race, is indicated by the orange horizontal line. It can be observed that most games in which the pilot set a qualifying time were toward the end of phase III of training, which continued until the Cybathlon finals. The best performance of the pilot (214 s) was also attained during phase III. We also examined the trend in performance across the training period and the impact of training on performance. The linear trend of the pilot's performance, denoted by the black dashed line, across the entire training indicates a moderate impact of BCI training (*r* = −0.1598), as measured via Pearson's correlation coefficient. The linear fit, however, is not statistically significant (*p* = 0.25) as the high variability between data points affects the estimation of the linear model. Nevertheless, it still indicates an inverse relationship between these variables.

**Figure 5:**
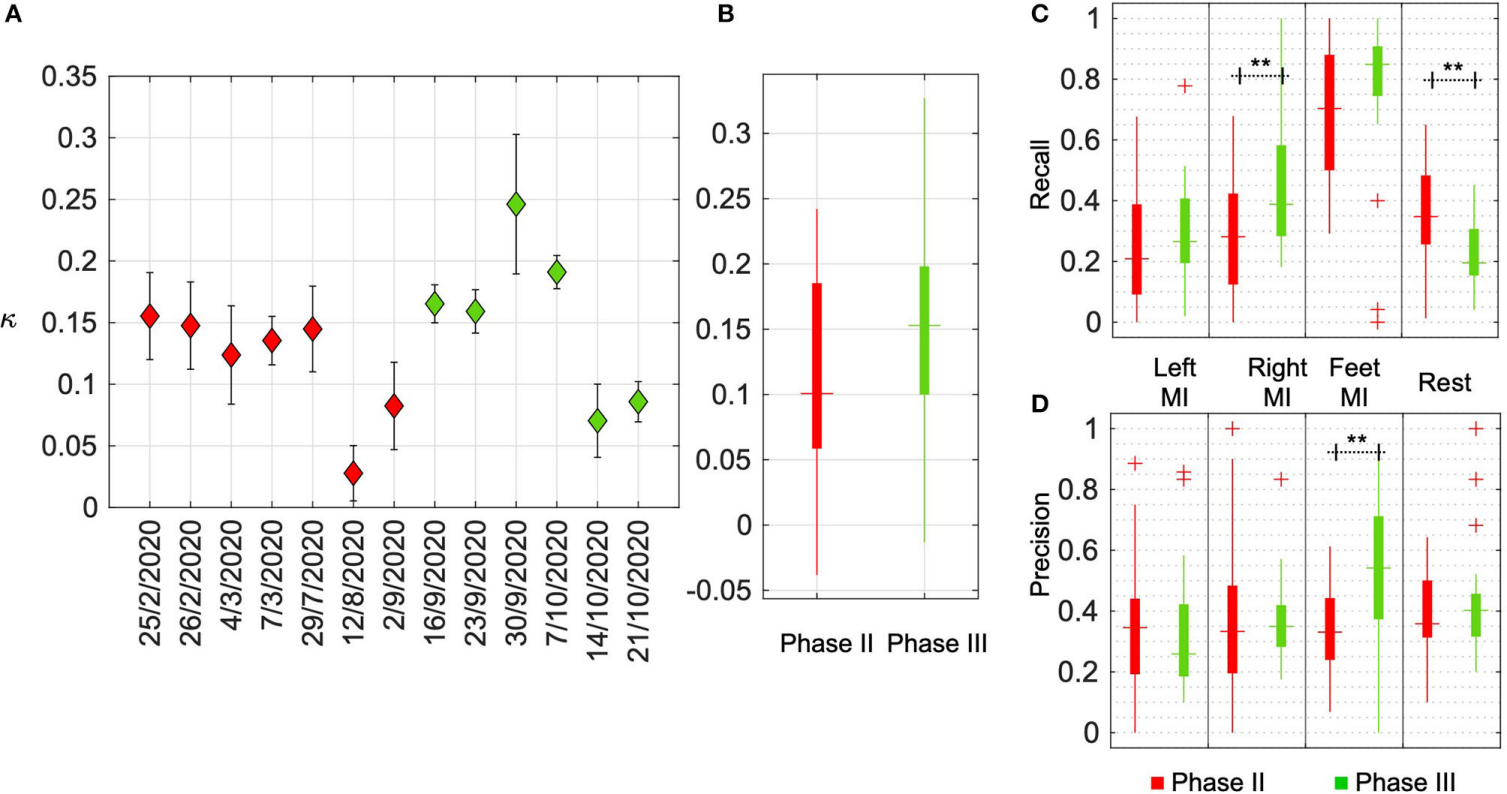
Evaluation of BCI performance using *κ*. **(A)** The figure shows *κ* for each session in phase II (in red) and phase III (in green). In each session, the pilot played four games and the data points indicate the mean and its standard error of *κ*. **(B)** The overall performance across all the sessions is displayed in red and green boxes indicating the performances in phase II and phase III, respectively. In each box, the horizontal dash indicates median value, the top and bottom edge of the box indicates the 25th and 75th percentiles, while the vertical line indicates the range of values. **(C,D)** The recall and precision values, respectively, across sessions for each of the MI classes. The columns indicate the metrics for each class, *ω* ∈ {Left MI, Right MI, Feet MI, Rest} and the boxplots show the change in these metrics from phase II to phase III. Columns marked with ** imply statistically significant differences with *p* < 0.001.

In order to further investigate the research objectives defined in this work, a BCI performance indicator that correlated with *τ*_*finish*_ was required. In this sub-section, the relevance of kappa (*κ*) as a standard metric to compare across the calibration and closed-loop BCI performances, across multiple calibrated models is presented. The relationship between *κ* and *τ*_*finish*_ is presented in Figure 4B. As expected, the linear regression between the two shows a significant linear fit (*p* < 0.001) and an inverse relation (*r* = −0.4262) between these two performance indicators. We present the variation in *κ* across game runs throughout our training period in Figure 5A. In each recording session, the pilot played four games and the data points in Figure 5A indicate the mean and its standard error of *κ*. In Figure 5B, the overall performance across all the sessions is displayed using red and green boxes that indicate performances in phase II and phase III, respectively. It can be observed in Figure 5A that, similar to *τ*_*finish*_, the improvement across the timeline was not significant. However, as indicated in Figure 5B, the median performance in phase III (*κ* = 0.15) was higher compared to phase II (*κ* = 0.10), although the difference between them was not statistically significant (*p* = 0.12). It can also be noted that the highest single game performance (*κ* = 0.33) was achieved in phase III.

In Figure 5A, it can also be observed that in both phases of training, while the performance holds steady for initial sessions, the performance falls as the training progresses. This may be accounted for by considering the increasing variability in EEG activity which could also be specific to each motor task. To study this further, the classification performance of each class (and hence the corresponding motor task), was investigated using the standard metrics of recall and precision. For each class, ω, recall indicates the ratio of samples correctly identified as belonging to ω to the total number of samples in that class. Precision indicates the ratio of samples correctly identified as belonging to ω to the total number of samples classified as this class. The recall and precision values across sessions are presented in Figures 5C,D. As observed in the recall values, the classification of Feet MI was consistently superior. The overall recall from phase II to phase III showed an improvement of 0.15 (*p* = 0.1) The precision values of Feet MI were also higher compared to other classes and it showed a significant (*p* = 0.0025) improvement from phase II to phase III. Across training sessions, the occurrence of samples mislabeled as Feet MI were reduced and this could be due to the pilot's ability to produce more distinct brain activity and the BCI model being able to identify them. For the Right MI, the recall value was observed to increase across time denoting that the subject was able to train to control this task. Subsequently, compared to phase II, a significant (*p* = 0.0091) increase in recall of Right MI was observed in phase III. However, the precision corresponding to this class was still low indicating that many other samples were misidentified as Right MI.

The recall and precision values for Left MI remained poor across the sessions. For Rest class, the recall deteriorated toward the end of the training period resulting in a significant (*p* = 0.0074) drop in phase III compared to phase II. While the precision indicates that in some sessions there were fewer samples incorrectly identified as Rest, the classifier failed to identify most of the samples belonging to this class. We also computed the weighted F-scores to quantify the overall performance across sessions. The F-scores were obtained as 0.31 and 0.34 for phase II and phase III, respectively, and the difference was not statistically significant (*p* = 0.16). This observation is similar to the *κ* computed across sessions.

### 3.2. Performance of Each Calibration Paradigm

One of the major goals of this study was to investigate the efficacy of different calibration paradigms. This research employed three different types of calibration paradigms, two of which used an open-loop BCI (*aC*, *gC*) but varied in GUI design, and the third one used a closed-loop BCI design (*gE*). To compare the performance we report the 10-fold cross-validation performance (*κ*_*cv*_) of the data recorded using each paradigm and a combination of the two open-loop paradigms. In each case, we further evaluate the performance (*κ*) of the models calibrated on the data recorded under each of these paradigms.

**Figure 6:**
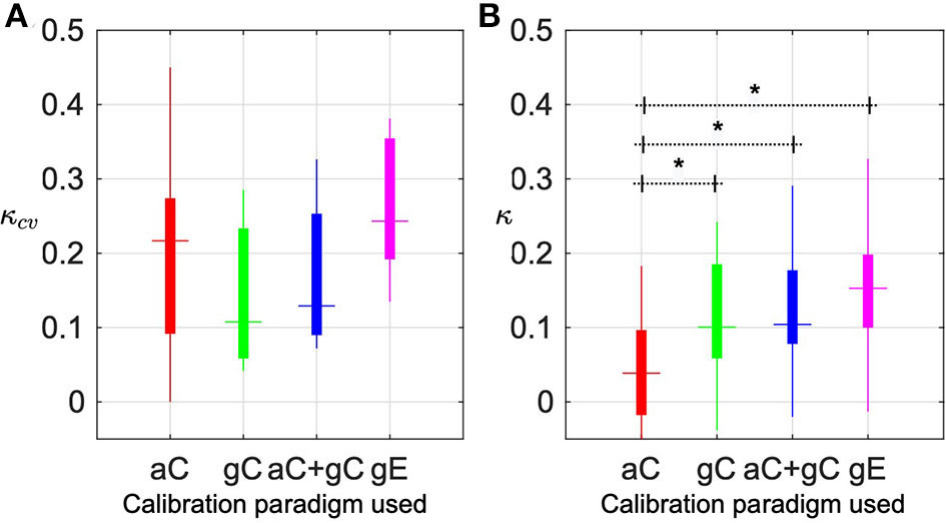
Performance of different calibration paradigms. **(A)** Box plots present the cross-validation performance, *κ*_*cv*_, across seven sessions, using arrow-based calibration (*aC*), game-based calibration (*gC*) and combined *aC* + *gC* and six sessions using game-based evaluation (*gE*). In each box, the horizontal dash indicates median value, the top and bottom edges of the box indicate the 25th and 75th percentiles and the vertical line indicates the range. **(B)** The boxplots represent online game performances using BCI, calibrated using each of these paradigms. The evaluation is based on four game runs in each of the seven sessions, for *aC*, *gC*, and combined *aC* + *gC* and six sessions for *gE*. Columns marked with * imply statistically significant difference (*p* < 0.05).

The results are presented in Figure 6. The box plots in Figure 6A present the cross-validation performance, *κ*_*cv*_, across seven sessions, using *aC*, *gC*, and combined *aC* + *gC* and six sessions using *gE*. In each box, the horizontal dash indicates median value, the top and bottom edges of the box indicate the 25th and 75th percentiles and the vertical line indicates the range. Based on *κ*_*cv*_, the closed-loop game based calibration, *gE* (*κ*_*cv*_=0.24), offered a slightly better median performance than conventional *aC* (*κ*_*cv*_=0.22). The difference, however, was not significant (*p* = 0.44). Compared to open-loop game based design, *gC* (*κ*_*cv*_=0.11), the closed-loop game design, *gE* elicited higher median kappa, however, the difference in performance was not statistically significant (*p* = 0.07).

The boxplots in Figure 6B, are online game performance using BCI, calibrated using each of these paradigms. The evaluation is based on four game runs in each of the seven sessions, for *aC*, *gC*, and combined *ac* + *gC* and six sessions for *gE*. In this study, to compare the closed-loop BCI performance using different models, we implemented a pseudo-online BCI pipeline. Hence, the performance is estimated for different models using the same recorded datastream from the experimental sessions, without fresh experiments with the pilot. Therefore, they do not take into account the subjective brain activity modulations that could happen during a real-time implementation of each of the models individually. The online performance shows a noticeable inversion in the trend compared to the cross-validation performance. The BCI model trained on *gE* resulted in a very significant (*p* = 8*e* − 4) superior performance (*κ* = 0.15), compared to conventional *aC* (*κ* = 0.04). The results from *gC* trained BCI model was *κ* = 0.10, which is again significant (*p* = 3.7*e* − 4) compared to *aC*. In both the results, a combination of both open-loop paradigms (*ac* + *gC*), showed comparable performance (*κ*_*cv*_ = 0.13 and *κ* = 0.10) to *gC*.

### 3.3. Impact of Recalibration in Performance

**Figure 7:**
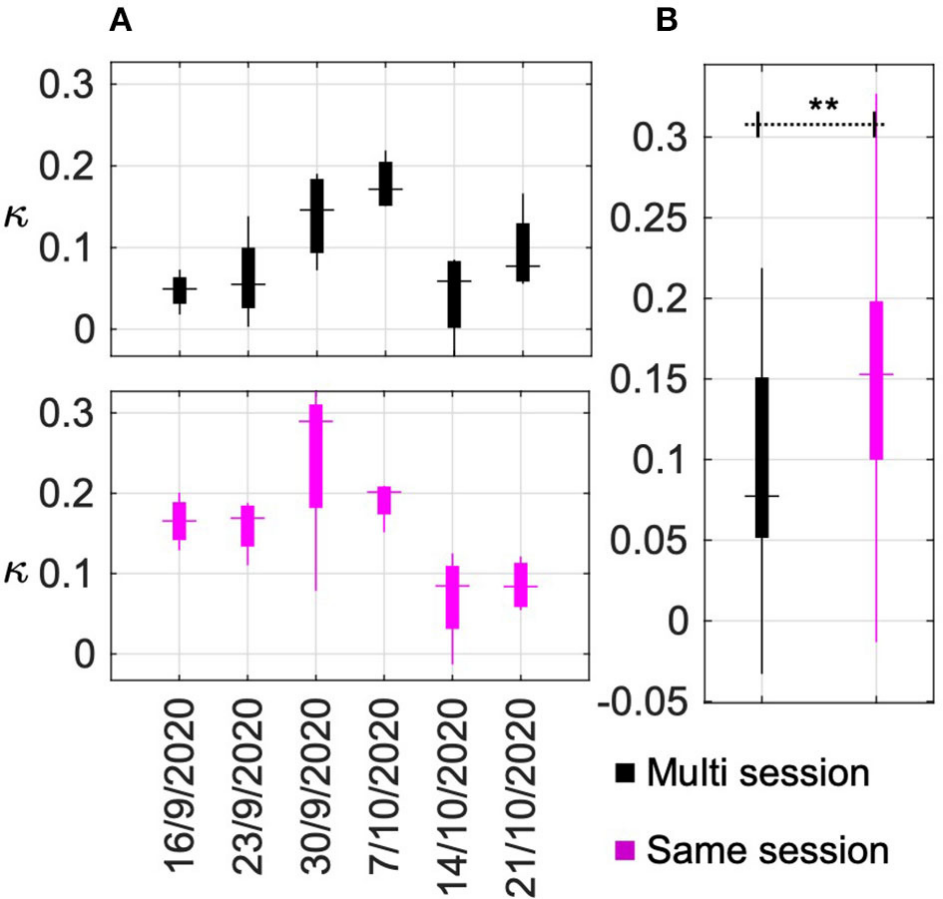
BCI performance with recalibration. **(A)** BCI performance across six sessions in phase III using “multi-session” and “same-session” models. Each box corresponds to the performance across the four game runs in each session. The horizontal dash indicates the median value, the top and bottom edges of the box indicate the 25th and 75th percentiles and the vertical line indicates the range. **(B)** Summary of the overall performance across all game sessions in phase III for the “multi-session” and “same-session” models. Columns marked with ** imply statistically significant difference with *p* < 0.001.

Another BCI design parameter that was evaluated in this research is whether a zero-calibration approach using a pre-trained model, trained on multiple sessions of data could be used for game evaluation on a separate game run. To evaluate this, a “multi-session” data pool was created using all the data recorded over the seven sessions during phase II. The classification performance of this multi-session data was determined by a 10-fold cross-validation. The average four-class classification accuracy and kappa value across the 10-folds was 45.74 ± (3.85)% and 0.28 ± (0.05)%, respectively. This data was then used to calibrate a BCI model. In each session of phase III, out of the eight game runs, the first four were used to calibrate a “same-session” BCI model. Using these two models, the last four runs in every session of phase III were evaluated. The performance across six sessions in phase III using “multi-session” and “same-session” models are presented in Figure 7A. Each box corresponds to the performance across the four game runs in each session. The horizontal dash indicates the median value, the top and bottom edges of the box indicate 25th and 75th percentiles and the vertical line indicates the range. The overall performance across all game sessions in phase III is summarized in Figure 7B. It can be observed that the performance of the models calibrated on the same day (*κ* = 0.15) offered significantly (*p* = 0.0081) higher game performance compared to a pre-trained model (*κ* = 0.08).

Since engagement and motivation are subjective factors, quantitative ratings were obtained from the pilot on all the experiment sessions. The pilot was asked to rate his mental and physical readiness on a scale of 1–5 (Alertness: 1 = very sleepy, 2 = sleepy, 3 = neither sleepy nor alert, 4 = alert, 5 = extremely alert and Physical tiredness: 1 = very tired, 2 = tired, 3 = neutral, 4 = fresh, 5 = lively, energetic) before and after each of the calibration runs. As expected, the monotonous design of *aC* resulted in a drop in the mental (*δ* = −0.14) and physical (*δ* = −0.43) states. The closed-loop game design, *gE*, however increased the alertness level of the pilot (*δ* = +0.17), but still caused a drop in energy level (*δ* = −0.17). The pilot's alertness level after *gC* increased (*δ* = +0.14), similar to *gE*. Unlike *gE*, it was also seen to increase the energy level of the pilot (*δ* = +0.14). The results presented in Figures 6, 7 indicate that a better BCI performance may be achieved with the help of (1) a calibration interface that offers active user engagement by real-time feedback, using a closed-loop protocol (*gE*) over an open-loop protocol (*aC* and *gC*) and closely resembles the end application; and (2) calibrating the BCI on the same day prior to evaluation so as to minimize variability between BCI model and the data for evaluation.

### 3.4. Analysis of Brain Activation Patterns

#### 3.4.1. Comparison of Dissimilarity Patterns Associated With Both Hands and Feet Classes

Sessions 5 and 6 used Both Hands as the fourth MI class and resulted in *κ*_*cv*_ = −0.008. Sessions 9 and 10 used Both Feet as the fourth MI class and resulted in *κ*_*cv*_ = 0.3333. Thus, multi-class classification with Both Feet MI was much better than that of Both Hands MI. We further investigated the neurophysiological signals to verify the discriminative ability of the evoked brain patterns corresponding to the fourth class vs. the other three classes. Dissociability of classes was assessed by comparing the pairwise differences between Both hands class, and the remaining three classes (Both hands vs. Left, Right and Rest), with the pairwise differences between Feet class, and the remaining three classes (Feet vs. Left hand, Right hand and Rest). The mean dissociability and its standard deviation were: M_BothHands_ = 0.49, SD_BothHands_ = 0.17; M_Feet_ = 0.68, SD_Feet_ = 0.21. Wilcoxon Rank Sum test revealed that the Feet class exhibited higher dissociability in comparison to the Both hands class (*p* = 0.03). Thus, this analysis further confirmed the comparatively higher discriminative ability of the Feet class as compared to Both hands as also observed from the classification performance above. We therefore opted to proceed with the Feet class as the fourth MI class.

#### 3.4.2. Evolution of Patterns Across Different Paradigms

**Figure 8:**
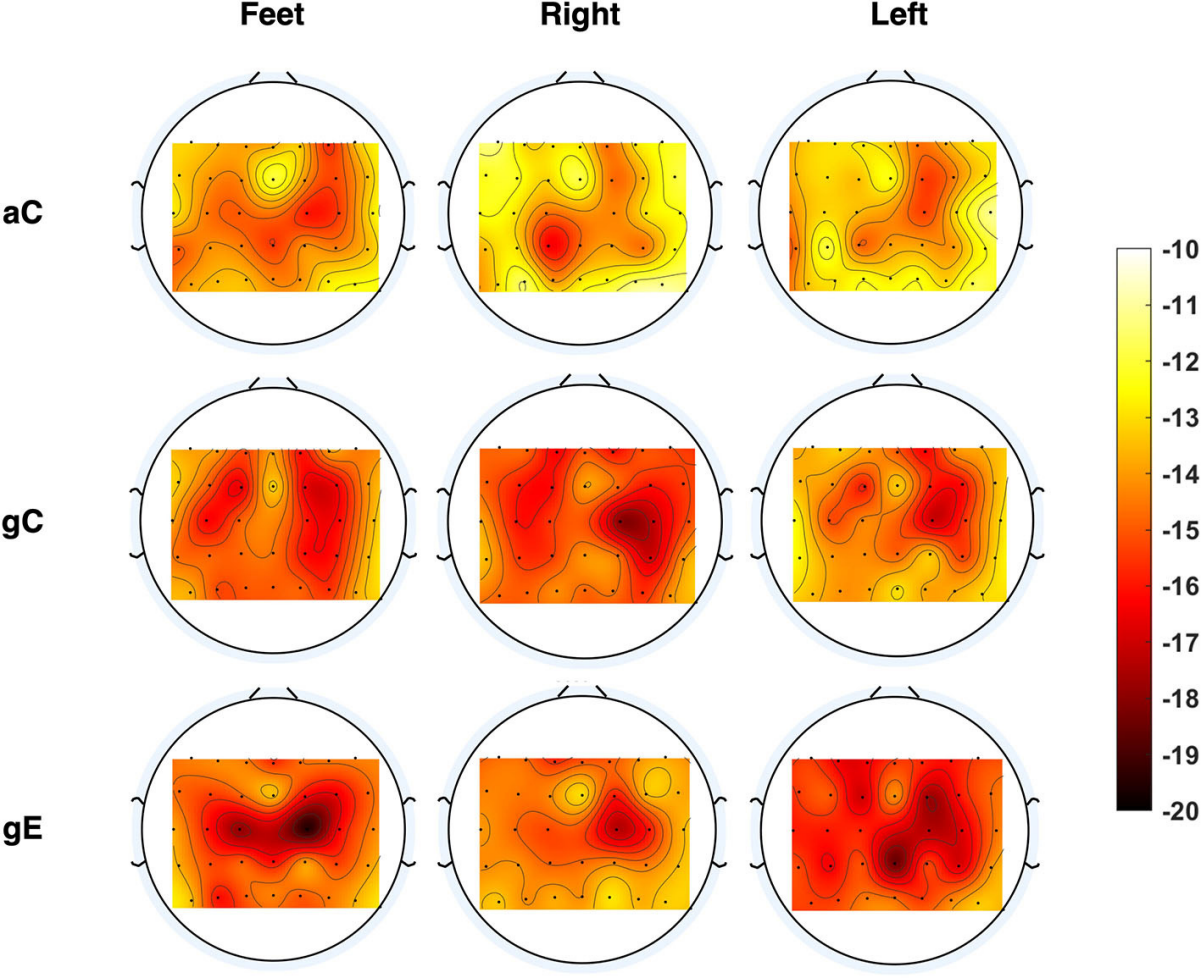
Evolution of brain activity. Topoplots showing brain activation patterns computed as the relative power (in dB) in the mu band (8–12 Hz) for the arrow-based calibration (*aC*), game-based calibration (*gC*), and the online games (*gE*) for Session 17 for the pilot in phase II.

As mentioned earlier, we first compared the strength of neural activations across the different kinds of paradigms. Figure 8 shows the topoplots in the mu band (Neuper et al., 2006) for one exemplary session, namely Session 17, from phase II for each *aC, gC*, and online game sessions (*gE*) for the three MI classes, using the channels above the motor and sensorimotor regions. While the *gE* seems to show stronger relative mu power depression and also more localized to the sensorimotor area, the same is seen to be weaker and much more diffused across a larger, widespread brain region in aC. Similar characteristics were also observed for other sessions. Similarly, Supplementary Figure 1 shows that the EEG channels over the sensorimotor region in phase III using *gE* were more discriminative whereas the peripheral channels seem to contribute more discriminative information to phase II using *gC*. A similar observation can also be made from the FBCSP topoplots shown in Supplementary Figure 2.

## 4. Discussion

This paper presented a long-term evaluation of BCI performance including one tetraplegic pilot. The results are based on data recorded during a period of 1 year and 10 months in which the pilot prepared to participate in the Cybathlon 2020 BCI race. Results demonstrated how certain BCI design considerations such as the BCI interface design, pilot training and BCI calibration strategies impact the overall BCI performance as well as the practical usability and end-user acceptance of BCI application.

Taking into account the need to enable and motivate the pilot to elicit distinct brain activities that are critical to control a BCI application, the present research investigated the BCI performance under different calibration paradigms varying in the presentation of instructions, feedback, and frequency of calibration of BCI. With regard to the visual interface used for the calibration experiments, the results indicated that a closed-loop game design that is similar to the end application offered the best median performance and user engagement, compared to traditional interfaces as well as other open-loop designs. The results in Figures 6A,B demonstrated that while the traditional MI design such as *aC* may perform better in a cross-validation analysis, a model calibrated on this data failed in a closed-loop game performance such as the Cybathlon BrainDriver game. The additional elements in a real-time BCI game environment may elicit different responses in the user's brain compared to the ones elicited by a simple visual interface. In case of *gC*, it can be observed that the online performance is comparable to that of a *gE* calibrated model. But at the same time *gC* reported the lowest *κ*_*cv*_ across all the paradigms. As indicated in section 3.3, the subjective assessment scores of the pilot's mental and physical readiness showed that he experienced greater engagement during *gE* as compared to other protocols. Even though, *gE* reported the best user engagement and cross-validation performance, this was not fully reflected in the online performance of *gE* calibrated model whose performance was higher, yet not statistically significant compared to *gC*. This may be explored in the future with the help of adaptive techniques to minimize the difference between calibration and evaluation datasets. Nevertheless, the results on subjective assessment and overall median performance provided evidence on the apparent advantages of a *gE* based calibration protocol. Several studies on BCI have reported the need to focus on the user-centric aspects of training protocols (Lotte et al., 2013; Chavarriaga et al., 2017; Roc et al., 2020) and user satisfaction/acceptance as a critical factor in long-term practical BCI applications (Holz et al., 2015). This study and the presented results fills these existing gaps in BCI and demonstrates the impact of calibration protocols in BCI.

We also presented brain activation maps for different paradigms, demonstrating the ability of the proposed closed-loop calibration paradigms (*gE*) in producing stronger and more localized EEG patterns over conventional paradigms. In Figure 8, it can be seen that the strength of the relative mu power suppressions followed the order *gE*>*gC*>*aC* (in dB) for most cases. We further visualized the CSP patterns from the two phases of training and then quantified the relationship between the discriminative power of various EEG channels and the overall classification performance. From the Supplementary Figures 1, 2, it is apparent that phase III includes correlated channels with the BCI performance around the sensorimotor region as compared to more laterally located areas in phase II. Figure 6 shows that *gE* resulted in the highest median classification performance as compared to the other calibration sessions. On the basis of these results, we postulate that adopting *gE* to recalibrate our classification models indeed seemed to improve the quality of the FBCSP filters obtained. As noted in Figures 5, 6, the improvement in classification performance in phase III also seemed to be correlate with the above observations in brain activation patterns for the different calibration paradigms. Thus, adopting the proposed closed-loop recalibration strategy may have also helped to further improve the median classification performance observed. Prior works in the literature have shown that different subjects may have varying brain spatial activation patterns during MI tasks, which have been said to increase the inter-subject and intra-subject variability of BCI decoders (Saha et al., 2017; Saha and Baumert, 2020). Even though our study only constitutes a single subject, we can see such variability in our results as well.

Past studies in literature have mentioned that several factors could be considered to enhance user-engagement in a BCI system (Škola et al., 2019; Roc et al., 2020). These include using calibration protocols that are closer to the end-application that the subject will use and using active feedback to inform the subject about his BCI performance. In this study, we were able to use some of these recommendations and observed the aforementioned improvement in user-engagement as enabled by moving from *aC* to *gC* in phase II and subsequently to *gE* in phase III. This also seemed to be correlated with the increasing strength of neural activation patterns seen in this work. Thus, our observations from the behavioral results may be correlated with the response of the underlying neural mechanisms to the various changes in training paradigms. On the basis of the trends in BCI performance as well as brain activation patterns for the different calibration paradigms, we suggest that it may be helpful to recalibrate the model to the data that are more closely indicative of the final task that needs to be performed by the subject. These observations seem to be in line with recent review studies in literature (Škola et al., 2019; Roc et al., 2020). However, it is also to be noted that while user-engagement may be one of the possible explanations, there might be other co-existing factors (such as role of feedback modality, etc.) leading to the above observations. Thus, future efforts should be expended toward investigating these factors in greater detail using systematic case-control studies.

Although our results seemed encouraging, our study is not devoid of limitations. Firstly, we noticed that the pilot's brain activity seemed to be undergoing continuous evolution throughout the training period across all phases. This can also be seen in Supplementary Figure 1 wherein there appear to be different groups of channels that are more discriminative (i.e., have higher Fisher Ratios) across the various sessions. We also noticed that there was major inter and intra-session variability in the EEG data. Although this is a known limitation of EEG data in general, this made it extremely challenging to use a common model across multiple sessions while maintaining usable classification performance. In this work, we chose to address this issue by recalibrating the classifier model to the same day's data in phase III. Our results show that it is indeed important to account for this continuous variation in EEG data for a usable BCI system and to maintain sustained engagement from the user. Future efforts may be driven toward more sophisticated approaches to address this issue such as using adaptation or transfer learning approaches. Because of such factors we noticed that while the proposed training paradigm was successful in keeping the user engaged and maintain reasonable classification accuracy, a significant learning effect was only seen for some MI tasks while only a weak trend, if present at all, could be seen for others. Moreover, the rules of Cybathlon BCI Race required teams to include appropriate artifact removal steps in the real-time classification pipeline. Since we desired to minimize the computational overhead added by the preprocessing and artifact removal steps to the BCI pipeline while providing high quality clean data to the BCI decoder, we chose to fix the number of channels marked for artifact removal to two. This number was empirically determined by analysing the effect of this parameter on the classification performance and amount of artifact removed. We found that this strategy was capable of identifying and removing artifacts, if present, satisfactorily as shown in this work. However future efforts may be devoted to develop more efficient methods that may also be able to detect the presence of an artifact prior to employing the artifact removal steps. This may avoid removing some important brain-related signals when there is little or no artifact in the signal. Dynamically adapting the number of channels to be corrected for artifacts may further help to produce higher performing BCI decoders. Overall, the above shows that there is still a lot of room for future studies to improve the current state of art in long term longitudinal training of SCI patients using a BCI system.

Lastly, we would also like to highlight that given the chain of calibration protocols used for the longitudinal training of our pilot to address his constantly evolving brain patterns, a few confounding factors also need to be kept in mind. Within a session the time duration and cue presentation was slightly different, which might have affected the pilot's mental state and workload. However, we tried to keep a track of the pilot's mental and physical fatigue by taking his feedback at regular intervals. While phases I and II started with *aC*, phase III directly started with *gE*. Thus, the choice of including the former may also have a role to play in the overall performance of the pilot on a given session, as *aC* was adjudged to be the most exhaustive and monotonous calibration protocol by the pilot. However, irrespective of the calibration phase, as mentioned earlier, we tried to keep an account of his mental workload by taking regular feedback. While comparing the performances of the calibration paradigms *aC, gC*, and *gE*, the total number of trials per class were slightly different, as indicated in Figure 2A. However, even though *gE* contained the fewest trials overall, it still seemed to perform better than conventional paradigms. Although we tried to increase the training data for *gE* by using more games for developing the model, this did not result in further improvement in classification performance on the remaining games during the pseudo-online analysis.

To conclude, in this study, we proposed to use an online BCI system, providing real-time feedback for the purpose of long term training of a tetraplegic SCI user. We chose to train the pilot directly on the game which was designed for the virtual BCI race that the pilot was supposed to participate in for Cybathlon 2020. Our results showed that by moving from a conventional offline, open-loop calibration paradigm to a real-time online calibration paradigm with continuous feedback using a graphical user interface that closely depicted the ultimate task to be performed by the user helped improve the quality of the BCI classifier as well as produce more discriminative brain activity patterns from the pilot. Neurophysiological evidence obtained suggested that improvement in behavioral characteristics of the pilot's game profile (such as the classification performance of the BCI and the subjective indices of user-engagement and fatigue) were underpinned by reorganization of neural mechanisms to produce more discriminative patterns in response to the proposed calibration methodology.

## Data Availability Statement

The datasets presented in this study can be found in online repositories. The names of the repository/repositories and accession number(s) can be found at: http://hdl.handle.net/20.500.11850/458693 (https://doi.org/10.3929/ethz-b-000458693) at ETH Research Collection.

## Ethics Statement

The studies involving human participants were reviewed and approved by ETH Zurich Ethics Committee (EK 2019-N-01). The patients/participants provided their written informed consent to participate in this study. Written informed consent was obtained from the individual(s) for the publication of any potentially identifiable images or data included in this article.

## Author Contributions

NR, RL, TC, EM, NW, and CG conceived the article's narrative. NR, TC, EM, and FD provided the analysis and BCI engine. NR, TC, and RL wrote the manuscript text and prepared the figures. PK and EM collected the data. All authors read, corrected, and approved the final manuscript.

## Conflict of Interest

BrainProducts has provided EEG equipment and Rehaklinik Zihlschlacht has financially supported the CYBATHLON BCI team for participating in the CYBATHLON 2020 event. The authors declare that the research was conducted in the absence of any commercial or financial relationships that could be construed as a potential conflict of interest.

## References

[B1] Abiri R. Borhani S. Sellers E. W. Jiang Y. Zhao X. (2019). A comprehensive review of EEG-based brain-computer interface paradigms. J. Neural Eng. 16:011001. 10.1088/1741-2552/aaf12e 30523919

[B2] Ang K. K. Chin Z. Y. Wang C. Guan C. Zhang H. (2012). Filter bank common spatial pattern algorithm on BCI competition IV datasets 2a and 2b. Front. Neurosci. 6:39. 10.3389/fnins.2012.00039 22479236 PMC3314883

[B3] Ang K. K. Chin Z. Y. Zhang H. Guan C. (2008). “Filter bank common spatial pattern (FBCSP) in brain-computer interface,” in 2008 IEEE International Joint Conference on Neural Networks (IEEE World Congress on Computational Intelligence), Hong Kong, 2390–2397.

[B4] Blankertz B. Muller K.-R. Curio G. Vaughan T. M. Schalk G. Wolpaw J. R.. (2004). The BCI competition 2003: progress and perspectives in detection and discrimination of EEG single trials. IEEE Trans. Biomed. Eng. 51, 1044–1051. 10.1109/TBME.2004.826692 15188876

[B5] Blankertz B. Muller K.-R. Krusienski D. J. Schalk G. Wolpaw J. R. Schlogl A.. (2006). The BCI competition iii: validating alternative approaches to actual BCI problems. IEEE Trans. Neural Syst. Rehabil. Eng. 14, 153–159. 10.1109/TNSRE.2006.875642 16792282

[B6] Brunner C. Leeb R. Müller-Putz G. Schlögl A. Pfurtscheller G. (2008). BCI Competition 2008-Graz Data Set A. Institute for Knowledge Discovery (Laboratory of Brain-Computer Interfaces); Graz University of Technology, 1–6.

[B7] Chang C.-C. Lin C.-J. (2011). LIBSVM: a library for support vector machines. ACM Trans. Intell. Syst. Technol. 2, 1–27. 10.1145/1961189.1961199

[B8] Chaudhary U. Birbaumer N. Ramos-Murguialday A. (2016). Brain-computer interfaces for communication and rehabilitation. Nat. Rev. Neurol. 12:513. 10.1038/nrneurol.2016.113 27539560

[B9] Chaudhary U. Mrachacz-Kersting N. Birbaumer N. (2020). Neuropsychological and neurophysiological aspects of brain-computer-interface (BCI) control in paralysis. J. Physiol. 599, 2351–2359. 10.1113/JP278775 32045022

[B10] Chavarriaga R. Fried-Oken M. Kleih S. Lotte F. Scherer R. (2017). Heading for new shores! overcoming pitfalls in BCI design. Brain Comput. Interfaces 4, 60–73. 10.1080/2326263X.2016.1263916 29629393 PMC5884128

[B11] Foldes S. T. Weber D. J. Collinger J. L. (2017). Altered modulation of sensorimotor rhythms with chronic paralysis. J. Neurophysiol. 118, 2412–2420. 10.1152/jn.00878.2016 28768745 PMC5646191

[B12] He B. Yuan H. Meng J. Gao S. (2020). Brain-Computer Interfaces. Cham: Springer International Publishing, 131–183. 10.1007/978-3-030-43395-6_4

[B13] Hochberg L. R. Serruya M. D. Friehs G. M. Mukand J. A. Saleh M. Caplan A. H.. (2006). Neuronal ensemble control of prosthetic devices by a human with tetraplegia. Nature 442, 164–171. 10.1038/nature04970 16838014

[B14] Holz E. M. Botrel L. Kaufmann T. Kübler A. (2015). Long-term independent brain-computer interface home use improves quality of life of a patient in the locked-in state: a case study. Arch. Phys. Med. Rehabil. 96, S16–S26. 10.1016/j.apmr.2014.03.035 25721543

[B15] Leeb R. Friedman D. Müller-Putz G. R. Scherer R. Slater M. Pfurtscheller G. (2007). Self-paced (asynchronous) BCI control of a wheelchair in virtual environments: a case study with a tetraplegic. Comput. Intell. Neurosci. 2007:79642. 10.1155/2007/79642 18368142 PMC2272302

[B16] Lotte F. Bougrain L. Cichocki A. Clerc M. Congedo M. Rakotomamonjy A.. (2018). A review of classification algorithms for EEG-based brain-computer interfaces: a 10 year update. J. Neural Eng. 15:031005. 10.1088/1741-2552/aab2f2 29488902

[B17] Lotte F. Larrue F. Mühl C. (2013). Flaws in current human training protocols for spontaneous brain-computer interfaces: lessons learned from instructional design. Front. Hum. Neurosci. 7:568. 10.3389/fnhum.2013.00568 24062669 PMC3775130

[B18] McFarland D. Norman S. Sarnacki W. Wolbrecht E. Reinkensmeyer D. Wolpaw J. (2020). BCI-based sensorimotor rhythm training can affect individuated finger movements. Brain Comput. Interfaces 7, 1–9. 10.1080/2326263X.2020.1763060

[B19] McFarland D. J. Miner L. A. Vaughan T. M. Wolpaw J. R. (2000). Mu and beta rhythm topographies during motor imagery and actual movements. Brain Topogr. 12, 177–186. 10.1023/A:1023437823106 10791681

[B20] McFarland D. J. Wolpaw J. R. (2011). Brain-computer interfaces for communication and control. Commun. ACM 54, 60–66. 10.1145/1941487.1941506 21984822 PMC3188401

[B21] Müller-Putz G. R. Daly I. Kaiser V. (2014). Motor imagery-induced EEG patterns in individuals with spinal cord injury and their impact on brain-computer interface accuracy. J. Neural Eng. 11:035011. 10.1088/1741-2560/11/3/035011 24835837

[B22] Nam C. S. Jeon Y. Kim Y.-J. Lee I. Park K. (2011). Movement imagery-related lateralization of event-related (de) synchronization (ERD/ERS): motor-imagery duration effects. Clin. Neurophysiol. 122, 567–577. 10.1016/j.clinph.2010.08.002 20800538

[B23] Neuper C. Wörtz M. Pfurtscheller G. (2006). ERD/ERS patterns reflecting sensorimotor activation and deactivation. Prog. Brain Res. 159, 211–222. 10.1016/S0079-6123(06)59014-4 17071233

[B24] Nikolaus Kriegeskorte Marieke Mur P. B. (2008). Representational similarity analysis–connecting the branches of systems neuroscience. Front. Syst. Neurosci. 2:8. 10.3389/neuro.06.004.2008 19104670 PMC2605405

[B25] Novak D. Sigrist R. Gerig N. J. Wyss D. Bauer R. Götz U. Riener R. (2018). Benchmarking brain-computer interfaces outside the laboratory: the cybathlon 2016. Front. Neurosci. 11:756. 10.3389/fnins.2017.00756 29375294 PMC5768650

[B26] Perdikis S. Millan J. d. R. (2020). Brain-machine interfaces: a tale of two learners. IEEE Syst. Man Cybern. Mag. 6, 12–19. 10.1109/MSMC.2019.2958200

[B27] Perdikis S. Tonin L. Saeedi S. Schneider C. Millán J. d. R. (2018). The cybathlon BCI race: successful longitudinal mutual learning with two tetraplegic users. PLoS Biol. 16:e2003787. 10.1371/journal.pbio.2003787 29746465 PMC5944920

[B28] Perrin F. Pernier J. Bertrand O. Echallier J. (1989). Spherical splines for scalp potential and current density mapping. Electroencephalogr. Clin. Neurophysiol. 72, 184–187. 10.1016/0013-4694(89)90180-6 2464490

[B29] Pfurtscheller G. Brunner C. Schlögl A. Da Silva F. L. (2006). Mu rhythm (de) synchronization and EEG single-trial classification of different motor imagery tasks. NeuroImage 31, 153–159. 10.1016/j.neuroimage.2005.12.003 16443377

[B30] Pfurtscheller G. Guger C. Müller G. Krausz G. Neuper C. (2000). Brain oscillations control hand orthosis in a tetraplegic. Neurosci. Lett. 292, 211–214. 10.1016/S0304-3940(00)01471-3 11018314

[B31] Pfurtscheller G. Neuper C. (2001). Motor imagery and direct brain-computer communication. Proc. IEEE 89, 1123–1134. 10.1109/5.939829

[B32] Roc A. Pillette L. Mladenovic J. Benaroch C. N'Kaoua B. Jeunet C.. (2020). A review of user training methods in brain computer interfaces based on mental tasks. J. Neural Eng. 18:011002. 10.1088/1741-2552/abca17 33181488

[B33] Saha S. Ahmed K. I. U. Mostafa R. Hadjileontiadis L. Khandoker A. (2017). Evidence of variabilities in EEG dynamics during motor imagery-based multiclass brain-computer interface. IEEE Trans. Neural Syst. Rehabil. Eng. 26, 371–382. 10.1109/TNSRE.2017.2778178 29432108

[B34] Saha S. Baumert M. (2020). Intra-and inter-subject variability in EEG-based sensorimotor brain computer interface: a review. Front. Comput. Neurosci. 13:87. 10.3389/fncom.2019.00087 32038208 PMC6985367

[B35] Sajda P. Gerson A. Muller K.-R. Blankertz B. Parra L. (2003). A data analysis competition to evaluate machine learning algorithms for use in brain-computer interfaces. IEEE Trans. Neural Syst. Rehabil. Eng. 11, 184–185. 10.1109/TNSRE.2003.814453 12899269

[B36] Škola F. Tinková S. Liarokapis F. (2019). Progressive training for motor imagery brain-computer interfaces using gamification and virtual reality embodiment. Front. Hum. Neurosci. 13:329. 10.3389/fnhum.2019.00329 31616269 PMC6775193

[B37] Tangermann M. Müller K.-R. Aertsen A. Birbaumer N. Braun C. Brunner C.. (2012). Review of the BCI competition IV. Front. Neurosci. 6:55. 10.3389/fnins.2012.00055 22811657 PMC3396284

[B38] Wang W. Collinger J. L. Degenhart A. D. Tyler-Kabara E. C. Schwartz A. B. Moran D. W.. (2013). An electrocorticographic brain interface in an individual with tetraplegia. PLoS ONE 8:e55344. 10.1371/journal.pone.0055344 23405137 PMC3566209

