# Supplementary material for: Design Considerations for Long Term Non-invasive Brain Computer Interface Training With Tetraplegic CYBATHLON Pilot
Source: Front Hum Neurosci. 2021 Jun 15;15:648275. doi: 10.3389/fnhum.2021.648275 (PMC8239283; doi:10.3389/fnhum.2021.648275)
Supplement: Supplementary file 1 [file Data_Sheet_1.PDF]

Supplementary Material

1 EEG DATA ANALYSIS FOR COMPARING CALIBRATION PARADIGMS

1.1 Identification of discriminative channels

We verify the discriminative ability of FBCSP models, quantified using Fisher Ratios of each EEG channel correlated with the classification performance of the overall model across the various sessions. Fisher Ratios offer an index of discriminative information available in two or more classes (Kam et al., 2013; Ma et al., 2016). It is computed as the ratio between the within-class and the between-class variance. Thus, using the above analysis, we seek to get insights into the underlying neurophysiological mechanisms that may help corroborate our observations from the classification performance of our BCI model across the various sessions of training.

From Figure S1a, we identify the EEG channels which show the most discriminative information for the various classes (as quantified using the Fisher Ratios using the relative mu power) that also correlate with the BCI performance κ. The figure shows the topoplots of the correlation coefficients of Fisher Ratios and classification performance obtained from EEG channels for Phases II and III, respectively. Higher values indicate the strong correlation between channels with higer discriminative power and decoding performance. Channels with significant correlation ($p < 0.05$) are marked with a pink circle. It can be seen that the channels with higher correlation values in Phase II are more laterally located, while those in Phase III are near the sensorimotor region. However, it can also be noted in Fig. S1b and S1c that the groups of channels showing higher discriminative information vary across sessions. In spite of this, the most discriminative channels in Phase III can be seen to lie closer to the motor and sensorimotor regions as expected. On the contrary, those in Phase II can be seen to be more spread throughout and occupy regions beyond the typical motor regions. Lastly, we visualize and compare the CSP patterns of two exemplary models from Phases II and III.

1.2 Analysis of FBCSP Models

After investigating the brain activity patterns of the above paradigms, we next seek to verify that the FBCSP models developed on gE are in fact more discriminative than those developed on gC. For this purpose we first visually compare the FBCSP patterns corresponding to the two kinds of models, assuming that a higher performing model would have more discriminative rather than more diffused FBCSP patterns.

Fig. S2 shows the FBCSP patterns in various frequency bands. It can be seen that the CSP coefficients in Session 17 from Phase II are smaller and produce less localized contours than the model in Session 25 from Phase III. Also, for Session 15, many of the peaks are near the sensorimotor region as typically expected from MI tasks. Comparatively, for topoplots in Session 17, the peaks in the CSP filters seem to be more widespread towards the periphery of the head rather than the more centrally located sensorimotor region.

REFERENCES

Kam, T.-E., Suk, H.-I., and Lee, S.-W. (2013). Non-homogeneous spatial filter optimization for electroencephalogram (eeg)-based motor imagery classification. *Neurocomputing* 108, 58–68

Ma, Z., Tan, Z.-H., and Guo, J. (2016). Feature selection for neutral vector in eeg signal classification. *Neurocomputing* 174, 937–945

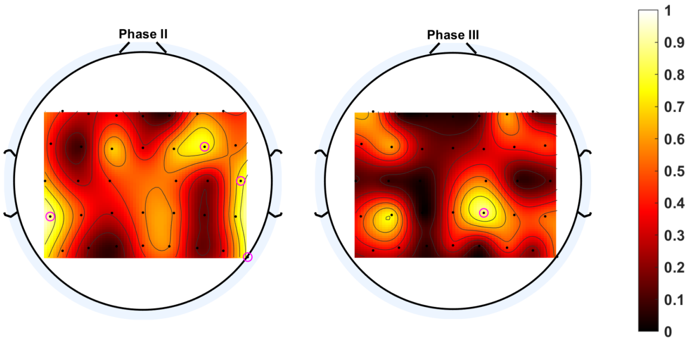

(a)

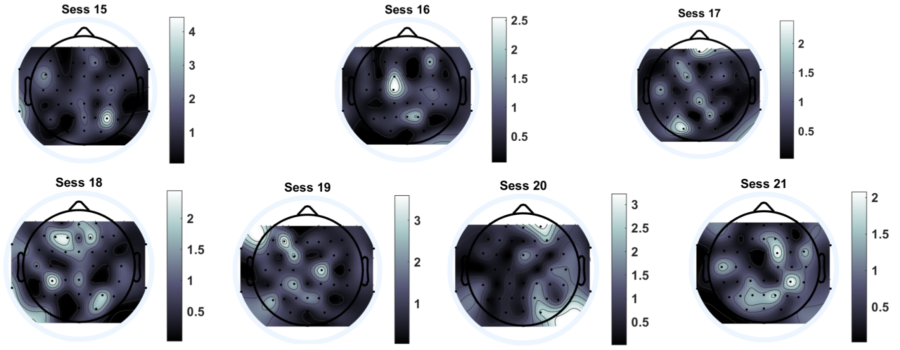

(b)

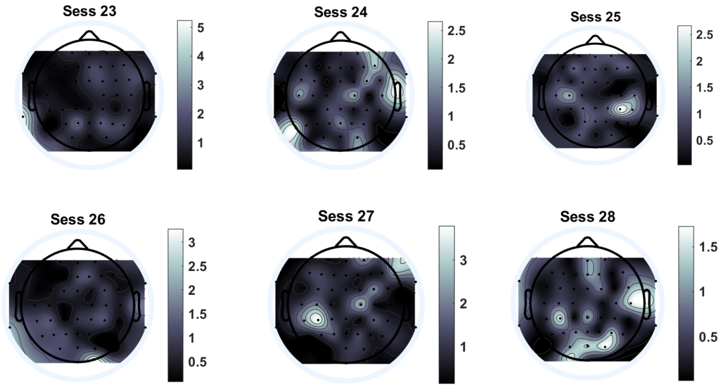

(c)

Figure S1. Correlation of Discriminative EEG Channels and Classifier Performance. (a) Topoplot showing the correlation of the Fisher Ratios of each EEG channel with the BCI classifier performance in Phase II and III. Channels that are significantly correlated (p <0.05) are annotated with a pink circle. Topoplots showing the Fisher Ratios of all EEG channels in (b) Phase II and (c) Phase III.

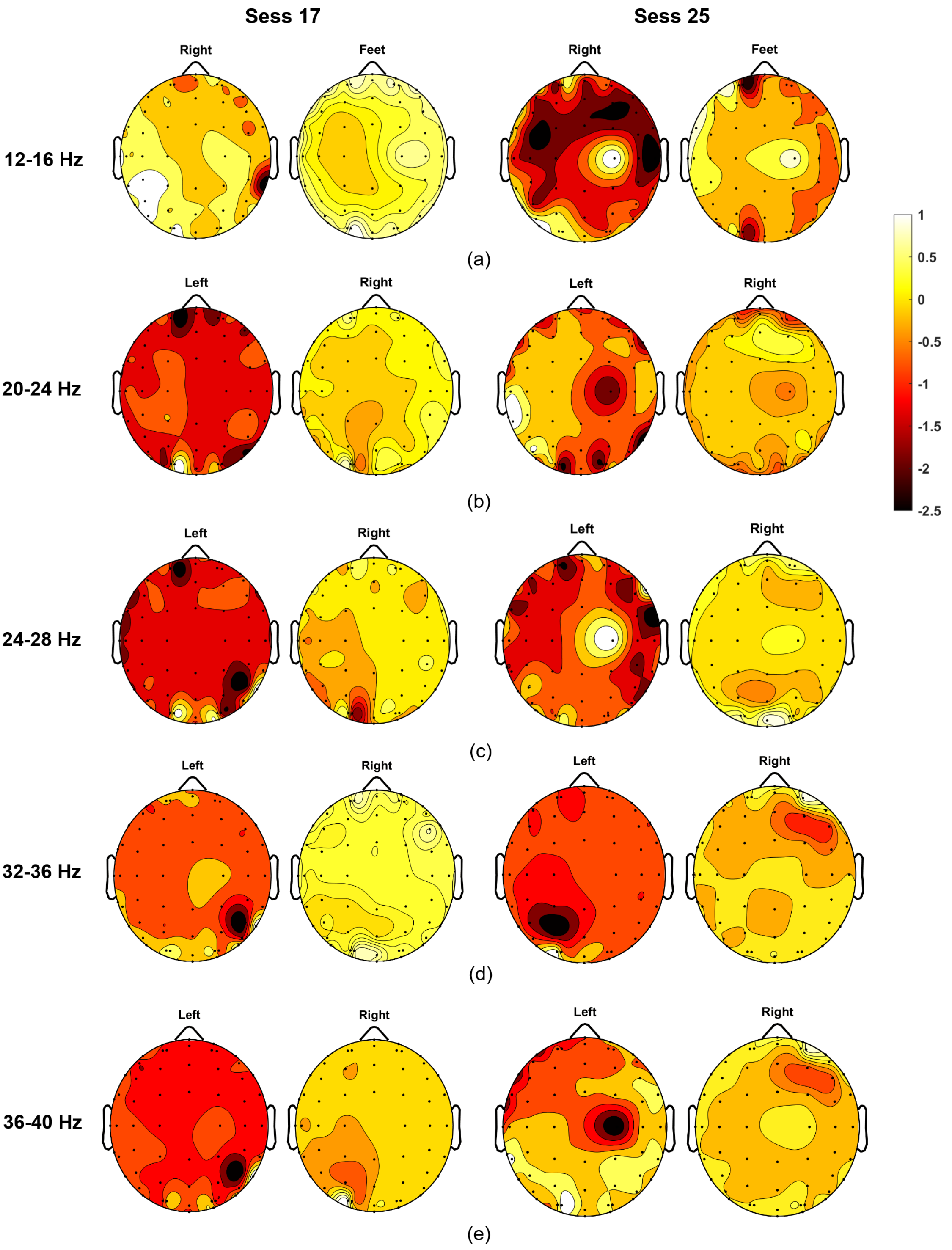

Figure S2. Comparison of FBCSP Patterns. Topoplots showing CSP pattern coefficients from models trained on Sessions 17 and 25 from Phases II and III, respectively in mu and beta bands. (a) shows exemplary CSP patterns for right vs feet MI in 12-16 Hz frequency band, (b) for left vs right MI in 20-24 Hz, (c) for left vs right MI in 24-28 Hz, (d) for left vs right MI in 32-36 Hz and (e) for left vs right MI in 36 - 40 Hz.
